# The Role of Psychological Well-Being in Weight Loss: New Insights from a Comprehensive Lifestyle Intervention

**DOI:** 10.1016/j.ijchp.2021.100279

**Published:** 2021-11-18

**Authors:** Boheng Zhu, Sara Gostoli, Giada Benasi, Chiara Patierno, Maria Letizia Petroni, Chiara Nuccitelli, Giulio Marchesini, Giovanni Andrea Fava, Chiara Rafanelli

**Affiliations:** a Department of Psychology “Renzo Canestrari”, University of Bologna, Italy; b Department of Psychological Medicine, Peking Union Medical College Hospital, Chinese Academy of Medical Sciences and Peking Union Medical College, China; c Department of Medical and Surgical Sciences, IRCCS-S. Orsola-Malpighi Hospital, University of Bologna, Italy; d Unit of Metabolic Diseases and Clinical Dietetics, IRCCS-S. Orsola-Malpighi Hospital, University of Bologna, Italy; e Department of Psychiatry, University at Buffalo, State University of New York, USA

**Keywords:** Lifestyle intervention, Obesity, Psychological well-being, Weight loss, Ex post facto study, Intervención en el estilo de vida, Obesidad, Bienestar psicológico, Pérdida de peso, Estudio ex post facto

## Abstract

**Background/Objective:**

Although the literature suggested that impaired psychological well-being (PWB) is associated with obesity, evidence on the role of PWB in weight outcomes is limited and inconclusive. This research aimed to investigate the joint role of PWB in achieving clinically significant weight loss (CWL; loss of 5% of the initial weight) through a comprehensive lifestyle intervention for obesity using a broad-based evaluation.

**Method:**

This study is a prospective cohort of 96 patients with obesity attending a comprehensive lifestyle intervention for weight loss. Data on weight, lifestyle, PWB, and distress, were collected before and after the intervention.

**Results:**

30.5% of the participants achieved CWL at the end of treatment. A more pronounced increase in autonomy (odds ratio = 0.80 [95% *CI*: 0.68, 0.93], *p* ≤ .01) and somatization (odds ratio = 0.83 [95% *CI*: 0.70, 0.98], *p* ≤ .05) from pre- to post-treatment were independently associated with a lower probability of CWL.

**Conclusions:**

Unbalanced dimensions of PWB, in particular exceedingly high autonomy, may contribute to a poor weight loss outcome. This study paves the way for the addition of psychotherapeutic strategies geared to euthymia in comprehensive lifestyle intervention.

## Abstract (es)

**Contexto/Objetivo:**

Aunque la literatura sugiere que el deterioro del bienestar psicológico (BP) está asociado con obesidad, la evidencia sobre el papel del BP en el peso es limitada. Se investiga el papel del BP en el logro de pérdida de peso clínicamente significativa (PPCS; pérdida del 5% del peso inicial) a través de una intervención integral sobre el estilo de vida con respecto a la obesidad.

**Método:**

Cohorte prospectiva de 96 pacientes con obesidad que asisten a una intervención integral sobre el estilo de vida para la pérdida de peso. Se recolectaron datos sobre peso, estilo de vida, BP y angustia, antes y después de la intervención.

**Resultados:**

El 30,5% de los participantes lograron pérdida de peso al final del tratamiento. Mayor aumento de autonomía (razón de momios = 0,80 [*IC* del 95%: 0,68, 0,93], *p* ≤ 0,01) y somatización (razón de momios = 0,83 [*IC* del 95%: 0,70, 0,98], *p* ≤ 0,05) de antes a después del tratamiento se asociaron de forma independiente con menor probabilidad de PPCS.

**Conclusiones:**

Las dimensiones desequilibradas del BP, en particular la autonomía excesivamente alta, pueden contribuir a una pérdida de peso insuficiente. Se allana el camino para añadir estrategias psicoterapéuticas orientadas a la eutimia en la intervención integral en el estilo de vida.

Obesity, defined as a Body Mass Index (BMI) ≥ 30 kg/m^2^ (World Health Organization, 2020), represents a serious global health issue (Paccosi et al., 2020). The prevalence of obesity has dramatically increased all over the world since 1975 (NCD Risk Factor Collaboration, 2017). According to a recent report of the World Health Organization (2020), 13% of the global adult population was affected by obesity in 2016. Obesity is highly associated with a chronic inflammatory state (Ellulu, Patimah, Khaza’ai, Rahmat, & Abed, 2017) that – on one hand – predisposes patients to a wide range of illnesses, such as type 2 diabetes, hypertension, hypercholesterolemia, cardiovascular diseases (National Institutes of Health, 2020), obstructive sleep apnea (Muscogiuri et al., 2019), and cancer (Avgerinos et al., 2019), thereby leading to a decrease in life expectancy (Prospective Studies Collaboration, 2009). On the other hand, a chronic inflammatory state may pave the way for the spread of pathogens, such as in the recent COVID-19 pandemic (Schett et al., 2020).

Though a variety of genetic, environmental, behavioral, and cultural factors may be involved in the development of obesity, an unhealthy lifestyle choice of “eating too much and moving too little” (National Institutes of Health, 2020) seems to be the commonest cause. Comprehensive lifestyle interventions aimed at modifying diet and physical activity habits together with behavioral strategies are considered as first-line treatment for the management of obesity and its comorbid conditions (Paccosi et al., 2020). Even though empirical data in the literature seem to support the efficacy of comprehensive lifestyle interventions in promoting weight reduction, a substantial variability across individuals’ responses has been found (Marek et al., 2017). Indeed, previous research considered as an indicator of positive outcomes even a minimal percentage of weight loss. However, it has been suggested that only a clinically significant weight loss (CWL), defined as a reduction of at least 5% of the initial weight (Donnelly et al., 2009), is associated with improvements in cardiometabolic risk factors, such as favorable changes in lipid profile and insulin sensitivity (Donnelly et al., 2009; Douketis, Macie, Thabane, & Williamson, 2005; Haire-Joshu & Hill-Briggs, 2018). For example, in the Look AHEAD trial (Look AHEAD Research Group, 2014), at the end of the intervention, more than 32% of the participants did not achieve a weight loss equal to or greater than 5% of the initial weight and, at 8-year follow-up, the majority of participants did not sustain CWL.

It has been argued that enduring lifestyle changes can only be achieved with a personalized approach that targets psychological well-being - PWB (Guidi et al., 2018). In the same vein, previous studies suggested that impaired psychological well-being (Elfhag & Rössner, 2005; Palmeira et al., 2010; Vallis, 2016) and high psychological distress (Escandón-Nagel et al., 2018; Sarwer et al., 2019) are associated with weight regain and may hinder healthy behavior promotion among patients with obesity. According to current literature, the construct of psychological well-being encompasses three main facets: hedonic well-being, eudaimonic well-being, evaluative well-being (Steptoe et al., 2015). The concept of hedonic well-being originates from the hedonic philosophical tradition that defines well-being as positive feelings and emotions in step with desires satisfaction (Diener, 2009). It refers to common everyday feelings or mood, such as happiness, sadness, anger, and stress (Kahneman et al., 2004). Hedonic well-being is not simply the opposite of negative feelings, as both hedonic well-being and negative feelings carry unique information about an individual's emotional state (Steptoe et al., 2015). By contrast, eudaimonic well-being advocates that well-being is achieved by realizing one's potentials in the pursuit of meaningful goals (Delle Fave et al., 2011). Drawing on Jahoda's work (1958), Ryff and Keyes (1995) have operationalized eudaimonic psychological well-being according to 6 dimensions such as autonomy, personal growth, purpose in life, environmental mastery, positive relations with others, and self-acceptance. Finally, evaluative well-being refers to people's opinions about the quality or goodness of their life and to what extent they are satisfied with their lives in general (Steptoe et al., 2015). Given that most of the studies considered psychological well-being as either the opposite or absence of psychological distress (i.e., depression, anxiety), or high quality of life, or positive self-esteem (Elfhag & Rössner, 2005; Palmeira et al., 2010; Vallis, 2016), to date evidence on the role of PWB in weight outcomes is limited and inconclusive.

To fill the gap in the literature, the objective of the present investigation was to explore the role of psychological well-being in clinically significant weight loss following a comprehensive lifestyle intervention program for obesity. As to this purpose, we performed a broad assessment of psychological well-being in patients with obesity, before and after the lifestyle intervention. We hypothesized that impaired psychological well-being dimensions would be associated with poor weight outcomes after the program.

## Method

### Study procedure and participants

This is a single-group prospective cohort study. Participants were consecutively recruited among a group of patients with obesity scheduled for an interdisciplinary behavioral lifestyle intervention program at the Center of Metabolism Diseases and Clinical Dietetics at Sant'Orsola Hospital in Bologna (Italy), between January 2018 and November 2019. Inclusion criteria were obesity (BMI ≥ 30) and age ≥ 18 years old. Patients were excluded if they (a) presented with a severe psychiatric illness and/or cognitive deficit; (b) were not fluent in Italian; (c) were pregnant within the last year; (d) underwent bariatric surgery within the last year; (e) took medications to reduce weight within the last year; (f) joined another lifestyle weight loss program within the last year. Participants were invited to complete a set of questionnaires at the beginning (T1) and the end (T2) of the comprehensive lifestyle intervention. Participation in the study was voluntary and documented with informed written consent, which was obtained for each patient. 95 participants (80.5% of the patients approached) who had completed both pre-and post-treatment assessments were included in this study. The present research has been approved by the Ethics Committee of Azienda Ospedaliero-Universitaria of Bologna, Italy.

### Comprehensive lifestyle intervention for obesity

The program was administered by a multidisciplinary team including physicians, dieticians, and a psychologist. The intervention consisted of 12 weekly sessions. Each session lasted about two hours and was held in a group setting (with a maximum of 20 participants). The main components of this program included general therapeutic education about obesity, lifestyle education (diet and exercise), and psychoeducation regarding motivation and maintenance of a healthy lifestyle.

In the opening session, a physician delivered a snapshot of obesity concerning its definition, etiologic factors, and complications. The second session was devoted to motivating the patients to make lifestyle modifications. The psychologist illustrated the stages of change (Prochaska & DiClemente, 1983) and discussed with the patients pro and cons of changing their lifestyle using a decisional balance sheet. Subsequently, the participants received 8 sessions of lifestyle education concerning diet and exercise. At first, a dietician provided the patients with the basics of healthy nutrition, which were based mainly upon the Meditteranean diet model, and encouraged them to make qualitative improvements. Secondly, the participants were trained how to use a structured diary, where they were asked to record their daily calories intake (by using a written list of foods caloric content) and expediture (i.e., basal metabolic rate and physical activity). Further, they were taught how to bank calories on a weekly basis, which allowed compensation between days, in order to help them managing calories in a flexible way. The penultimate session was held by a physician and dedicated to therapeutic education about bariatric surgery. Finally, a psychologist delivered a session focused on preventing relapses, in which the participants were trained how to identify prodromes of crisis related to weight regain and to use problem-solving techniques to promote and maintain healthy eating habits and physical activity.

### Instruments

Participants were evaluated at the beginning and the end of the behavioral lifestyle intervention. Medical history, along with sociodemographic data, including age, gender, marital status, education, and employment, were collected at baseline.

Hedonic well-being. The Symptom Questionnaire (SQ; Benasi et al., 2020; Kellner, 1987) is a 92-item self-rating scale for the assessment of psychological distress over a 1-week time interval. The SQ yields 4 main scales: Depression, Anxiety, Somatization, and Hostility. Each scale can be further divided into 2 subscales: one related to symptoms (i.e., depression, anxiety, somatization, and hostility) and the other to well-being (i.e., contentment, relaxation, physical well-being, and friendliness). Answers on each item are dichotomous (i.e., *Yes*/*No* or *True*/*False*). For each of the 4 main scales, a total score was calculated by integrating distress and well-being subscales; higher scores indicate greater psychological distress. The SQ demonstrated adequate clinimetric properties across various clinical settings (Benasi et al., 2020).

Eudaimonic well-being. The short version of the Psychological Well-Being scales (PWBs; Ryff & Keyes, 1995) is a 42-item self-rating questionnaire that evaluates 6 dimensions of psychological well-being: self-acceptance, positive relations with others, autonomy, environmental mastery, purpose in life, and personal growth. Respondents were asked to rate on a 6-point Likert scale the extent to which they agreed with each statement (from 1= *strongly disagree* to 6= *strongly agree*). Subtotal scores of each dimension, which may range from 7 to 42, were calculated separately. Higher scores indicate higher levels of psychological well-being in the corresponding dimension. The PWBs showed acceptable validity and reliability across different samples (Ryff, 2014).

Evaluative well-being. A straightforward question (“How do you rate the quality of your life”) derived from PsychoSocial Index (PSI; Piolanti et al., 2016; Sonino & Fava, 1998) was used to rate the quality of life on a scale ranging from 4 (*excellent*) to 1 (*awful*). The adequate clinimetric properties of PSI have been documented in different samples (Piolanti et al., 2016).

Weight. Body weight was measured to the nearest 0.1 kg on a standard balance beam scale with the participants in lightweight clothing. Stadiometer was used to measure height to the nearest 1.0 cm with the participants standing without shoes. Both body weight and height were used to calculate BMI. Clinically significant weight loss (CWL) was defined as at least a 5% weight loss from the initial body weight, which was deemed essential to produce adequate physical health improvement in obesity-related risk factors, such as adverse cardio-metabolic profile (Donnelly et al., 2009).

Lifestyle. The GOSPEL questionnaire (Giannuzzi et al., 2008) is a 32-item self-rating instrument for the assessment of diet habits and physical activity levels over a 6-month time interval. The instrument, which has been used in several studies in cardiac settings (Bernardini et al., 2020; Giannuzzi et al., 2008; Gostoli et al., 2016), includes 10 items on a Mediterranean diet that evaluate the frequency of consumption of specific categories of food (such as vegetables, fish, butter), on a scale ranging from 1 (*never or rarely*) to 4 (*every day*); scores on each item are summed to obtain a Mediterranean diet score ranging from 0 (*worst*) to 30 (*best*). Moreover, 3 additional items assess eating behaviors based on how often respondent eats regularly, slowly, and in a relaxed way, on a scale ranging from 1 (*never*) to 4 (*always*); scores on each item are summed to obtain an eating behavior score from 0 (*worst habits*) to 9 (*best habits*). Eight items assess physical activity. Specifically, 5 items evaluate how frequently respondents engage in specific physical activities (such as climbing stairs, walking, doing gym) on a scale from 1 (*never or rarely*) to 4 (*every day*), 2 items investigate the presence of additional physical activities, with yes/no answer options, and 1 item assesses the overall self-perceived level of physical activity on a scale ranging from 4 (*very high*) to 1 (*poor*). Scores on each of these items are summed to obtain a total physical activity score from 0 (*least active*) to 20 (*most active*).

### Data analyses

Data were analyzed by means of the Statistical Package for Social Science (SPSS, version 24). Chi-squared tests (for categorical variables) and Pearson's correlation (for continuous variables), were used to determine the bivariate relationships between sample characteristics at baseline and CWL (coded as CWL = 1, non-CWL = 0). Furthermore, paired sample *t*-tests were performed to evaluate changes from baseline, whereas Pearson's correlations to test the bivariate associations of changes in psychological well-being and lifestyle with CWL. For each patient, the changes in psychological well-being, body weight, and lifestyle were calculated by subtracting the post-treatment scores from the baseline scores (Δ variables = T1-T2). Effect sizes were estimated using Pearson's *r* (i.e., .10 small, .30 medium, and .50 large) and Cramer's *v* (i.e., .25 small, .50 medium, and .80 large) (Cohen, 1988).

Multivariate binary logistic regression models were used to determine the independent associations of psychological well-being factors with CWL (set as the outcome). Eudaimonic (PWBs), hedonic (SQ), and evaluative (PSI) well-being, were entered as predictors in logistic regression models provided that they showed a significant bivariate relationship with CWL (*p* ≤ .05). As to eudaimonic, hedonic, and evaluative psychological well-being, variables were analyzed as pairs (i.e., when scores changes from baseline to post-intervention were significant according to the bivariate analyses, they were included in the regression models, together with their respective baseline levels), given that the magnitude of change of each variable might be confounded by its baseline level. Multivariate analyses were launched according to two models: the first where psychological well-being variables were adjusted for relevant demographic and medical history data (*p* ≤ .05 according to the bivariate analyses); the second additionally encompassing lifestyle variables (including both baseline levels and scores change from baseline to post-intervention). This analysis plan allowed us to establish whether the association of psychological well-being with weight outcomes persisted even after adjusting for lifestyle factors.

The model quality was evaluated using the Hosmer‐Lemeshow *χ^2^* test (with significant *χ^2^* statistics indicating a poor model fit) and Nagelkerke *R^2^* (with values > .20 considered as acceptable amounts of explained variability) (Hosmer & Lemeshow, 2001).

Statistical power analyses were performed using G*Power 3.1. The results showed that at least 70 cases were necessary to reach a power of .80 to detect a small-to-medium effect size (odds ratio = 2.50) in logistic regression, adopting an alpha level of .05 (two-tailed). The significance level for all the statistical tests was set to *p* ≤ .05, two-tailed.

## Results

The baseline sociodemographic and clinical characteristics of the sample (*n* = 95) are presented in Table 1. In the overall cohort, the age of participants ranged from 22 to 77 years old, with a mean age of 55.63 years (*SD* = 10.80). Among them, 75.8% were female, 75.8% had a high school diploma or higher, 56.8% were employed, and 21.1% were living alone. 30.5% of the participants achieved clinically significant weight loss. The mean weight loss was -3.34 (*SD* = 4.36).

**Table 1:** Baseline sample characteristics and their bivariate relationships with clinically relevant weight loss (CWL).

Variables	Overall(*n* = 95)	CWL^a^(*n* = 29)	Non-CWL^b^(*n* = 66)	*p*	Effect size
Age (y), *M ± SD*	55.63 ± 10.80	54.66 ± 11.65	56.06 ± 10.48	.562	-0.06^c^
Female sex, *n*(%)	72 (75.8)	25 (86.2)	47 (71.2)	.116	0.16^d^
Education				.611	0.05^d^
Middle school or lower, *n(%)*	23 (24.2)	8 (27.6)	15 (22.7)		
High school or higher, *n(%)*	72 (75.8)	21 (72.4)	51 (77.3)		
Currently Employed	54 (56.8)	19 (65.5)	35 (53.0)	.258	0.12^d^
Living alone, *n*(%)	21 (22.1)	10 (34.5)	11 (16.7)	.054	0.20^d^
Cardiovascular diseases, *n*(%)	55 (57.9)	16 (55.2)	49 (59.1)	.722	0.04^d^
Diabetes, *n*(%)	28 (29.5)	8 (27.6)	20 (30.3)	.755	0.03^d^
Hyperchloremia, *n(%)*	36 (37.9)	11 (37.9)	25 (37.9)	.961	0.01^d^
Hypertension, *n(%)*	48 (50.5)	16 (55.2)	32 (48.5)	.595	0.06^d^
Smoking, *n(%)*	6 (6.3)	2 (6.9)	4 (6.1)	.892	0.01^d^
Body weight, *M ± SD*	101.50 ± 19.27	99.72 ± 17.52	102.28 ± 20.07	.555	-0.06^c^
BMI, *M ± SD*	37.43 ± 5.66	37.48 ± 5.11	37.40 ± 5.92	.953	0.01^c^
SQ (hedonic well-being)					
Anxiety, *M ± SD*	6.12 ± 4.22	7.50 ± 3.65	5.50 ± 4.34	**.033**	0.22^c^
Depression, *M ± SD*	5.96 ± 3.97	7.17 ± 4.26	5.42 ± 3.75	**.048**	0.20^c^
Somatization, *M ± SD*	9.66 ± 5.33	10.16 ± 5.10	9.45 ± 5.44	.553	0.06^c^
Irritability, *M ± SD*	5.50 ± 4.73	5.53 ± 4.95^c^	5.48 ± 4.66	.957	0.01^c^
PWBs (eudaimonic well-being)					
Autonomy, *M ± SD*	30.09 ± 6.16	29.31 ± 5.74	30.43 ± 6.35	.417	-0.08^c^
Environmental mastery, *M ± SD*	28.87 ± 6.28	28.33 ± 6.35	29.11 ± 6.28	.577	-0.06^c^
Personal growth, *M ± SD*	31.75 ± 5.65	30.83 ± 6.03	32.16 ± 5.47	.293	-0.11^c^
Positive relationship, *M ± SD*	31.57 ± 6.41	31.09 ± 6.81	31.79 ± 6.27	.626	-0.05^c^
Purpose of life, *M ± SD*	28.51 ± 5.18	28.45 ± 4.89	28.53 ± 5.34	.944	-0.01^c^
Self-acceptance, *M ± SD*	27.11 ± 6.75	25.85 ± 6.73	27.66 ± 6.73	.229	-0.12^c^
PSI (evaluative well-being)					
Quality of life, *M ± SD*	2.16 ± 0.77	1.95 ± 0.71	2.18 ± 0.78	.172	-0.14^c^
GOSPEL					
Physical activity, *M ± SD*	4.56 ± 2.77	4.78 ± 2.79	4.45 ± 2.77	.605	0.06^c^
Diet, *M ± SD*	19.10 ± 4.80	20.24 ± 5.77	18.57 ± 4.23	.122	0.16^c^
Eating behavior, *M ± SD*	4.78 ± 2.06	4.79 ± 1.93	4.77 ± 2.13	.974	<0.00^c^

*Note:* SQ = Symptom Questionnaire; PWBs = Psychological Well-Being scales; PSI= PsychoSocial Index; GOSPEL = GOSPEL scale for lifestyle characteristics; *SD* = Standard deviation.

Bold: *p* ≤ .05.

a Weight loss ≥ 5% of baseline weight

b Weight loss < 5% of baseline weight

c Pearson's *r*

d Cramer's *v.*

The changes in psychological well-being dimensions and lifestyle scores, from pre- to post-intervention, are displayed in Table 2. In the CWL-group, a significant improvement from baseline was observed in SQ somatization scale (*t* = 2.85, *p* = .008), whereas the score of SQ anxiety (*t*= -2.29, *p* = .026) and PWBs autonomy (*t* = -3.02, *p* = .004) significantly increased in the non-CWL-group. Besides, both groups showed significant improvements in lifestyle habits scores (*p* ≤ .05).

**Table 2:** Changes in psychological wellbeing and lifestyle variables and their bivariate relationships with clinically relevant weight loss (CWL).

Variables	CWL^a^ (*n* = 29)*M ± SD*	Non-CWL^b^ (*n* = 66)*M ± SD*	*p*^c^	Pearson's *r*
Change from baseline measure^d^				
SQ (hedonic well-being)				
Δ Anxiety	-0.41 ± 4	**1.10 ± 3.85**^e^	.086	-.18
Δ Depression	-0.47 ± 3.91	0.52 ± 3.00	.187	-.14
Δ Somatization	**-2.78 ± 5.25**^e^	-0.22 ± 4.43	**.017**	-.25
Δ Irritability	0.05 ± 3.57	0.42 ± 4.24	.688	-.04
PWBs (eudaimonic well-being)				
Δ Autonomy	-0.84 ± 5.45	**1.54 ± 4.14**^e^	**.022**	-.24
Δ Environmental mastery	-0.76 ± 4.02	0.27 ± 4.73	.312	-.11
Δ Personal growth	0.83 ± 5.55	-0.14 ± 4.85	.392	-.09
Δ Positive relationship	-0.59 ± 5.56	0.69 ± 4.52	.242	-.12
Δ Purpose of life	-0.66 ± 4.94	0.14 ± 4.63	.454	-.08
Δ Self-acceptance	0.69 ± 5.44	0.31 ± 6.07	.773	-.03
PSI (evaluative well-being)				
Δ Quality of life	0.07 ± 0.76	0.05 ± 0.78	.931	.01
GOSPEL				
Δ Physical activity	**1.09 ± 2.25**^e^	**1.08 ± 2.30**^e^	.991	<.00
Δ Diet	**1.29 ± 2.48**^e^	**1.01 ± 2.90**^e^	.648	.05
Δ Eating behavior	**1.14 ± 1.41**^e^	**0.82 ± 1.59**^e^	.358	.10

*Note:* SQ = Symptom Questionnaire; PWBs = Psychological Well-Being scales; PSI = PsychoSocial Index; GOSPEL = GOSPEL scale for lifestyle characteristics.

Bold: *p* ≤ .05.

a Weight loss ≥ 5% of baseline weight;

b Weight loss < 5% of baseline weight;

c Pearson correlation tests for bivariate relationships between variables and CWL;

d Δ variable= T2 (post-intervention)-T1 (baseline);

e *t*-tests for changes from baseline.

### Bivariate analyses

As illustrated in Table 1 and Table 2, CWL positively correlated with baseline SQ anxiety and depression, and negatively with changes in PWB autonomy and SQ somatization, from pre- to post-intervention.

### Multivariate analyses

Multivariate logistic regression analyses (Table 3) demonstrated that a greater baseline SQ somatization score (odds ratio = 0.85 [95% *CI*: 0.74, 0.99], *p* ≤ .05), as well as a more pronounced increase in SQ somatization (odds ratio = 0.81 [95% *CI*: 0.69, 0.94], *p* ≤ .01) and PWB autonomy (odds ratio = 0.82 [95% *CI*: 0.70, 0.95], *p* ≤ .01), were associated with a lower probability of achieving CWL (Model 1). After controlling for lifestyle factors (Model 2), the adjusted odds ratios were 0.83 [95% *CI*: 0.70, 0.98], *p* ≤ .05) for SQ somatization and 0.80 [95% *CI:* 0.68, .93], *p* ≤ .01) for PWB autonomy, whereas the association of baseline SQ somatization with CWL was not significant any more. A good model fit was found for each model: Model 1 (Hosmer‐Lemeshow *χ^2^* = 4.54, *p* = .805; Nagelkerke *R^2^* = .35); Model 2 (Hosmer‐Lemeshow *χ^2^*= 3.12, *p* = .926; Nagelkerke *R^2^* = .45).

**Table 3:** Multivariate logistic regression, reported as odds ratios (*95%CI*), of clinical relevant weight loss.

Outcome: CWL		
Variable	Model 1	Model 2^a^
SQ (hedonic well-being)		
Δ Somatization^b^	**0.81 [0.69, 0.94]**⁎⁎	0.83 [0.70, 0.98]*
Somatization baseline	0.85 [0.74, 0.99]*	n.s.
Anxiety baseline	n.s.	n.s.
Depression baseline	n.s.	n.s.
PWBs (eudaimonic well-being)		
Δ Autonomy^b^	**0.82 [0.70, 0.95]**⁎⁎	**0.80 [0.68, 0.93]**⁎⁎
Autonomy baseline	n.s.	n.s.
*R^2^*	.35^c^	.45^d^

*Note*: CI = confidence interval; CWL = Clinically significant weight loss; n.s. = Not significant; PWBs = Psychological Well-Being scales; SQ = Symptom Questionnaire.

⁎ *p* ≤ .05;

⁎⁎ *p* ≤ .01.

a Adjusted for lifestyle variables (both baseline levels and change);

b Δ variable= T1(baseline)-T2(post-intervention);

c Hosmer‐Lemeshow *χ^2^*= 4.54, *p*= .805;

d Hosmer‐Lemeshow *χ^2^*= 3.12, *p* = .926.

## Discussion

The present study supports the role of psychological well-being in achieving clinically significant weight loss (CWL) after multi-disciplinary lifestyle intervention for obesity. The main strength of this study is represented by its comprehensive evaluation of the construct of psychological well-being.

Concerning eudaimonic psychological well-being, the present study revealed that changes in autonomy were significantly and independently associated with CWL. Specifically, we found that a more prominent increase of autonomy scores, from pre- to post-intervention, was associated with a poorer weight reduction. Given the dimensional nature of autonomy (Carrozzino et al., 2019; Fava, 2016; Fava & Guidi, 2020; Guidi et al., 2018), on one hand, impaired autonomy could characterize an individual who is over-dependent on others. On the other hand, also excessive levels of psychological well-being can be detrimental, as suggested by literature (Gostoli et al., 2021; Rafanelli et al., 2016). Indeed, in the case of high autonomy, the individual might be over-confident about his/her skills and/or be unable to learn from others and to accept advice (Carrozzino et al., 2019; Fava, 2016; Fava & Guidi, 2020; Guidi et al., 2018). Over-confidence entails many psychological biases and misjudgments (Pronin et al., 2002), which can hamper recovery (Borland & Balmford, 2005). Hence, in our study, a pronounced increase in autonomy during the comprehensive lifestyle intervention might have led the patients to believe that managing obesity was not as challenging as they thought in the beginning, sustaining the dysfunctional idea that they no longer needed help and advice from their physicians. As a consequence, these distorted thoughts could have weakened patients’ coping skills to deal with the challenges in achieving a relevant weight loss. Further studies involving a control group are needed to consider autonomy and psychological well-being balance as mediators of the effect of lifestyle intervention on weight loss.

Besides, the improvement of somatization was strictly associated with CWL. This finding is in line with literature showing a decrease of pain and comorbid symptoms in patients with obesity who lost weight after joining lifestyle interventions (Razeghi Jahromi et al., 2019; Schrepf et al., 2017). It could be hypothesized that, among patients who attained a CWL, the reduction of somatization may be linked to the improved management of the medical comorbidities associated with obesity. These findings support the clinical implication of the CWL threshold and suggest that a substantial weight loss may be relevant to physical benefits. Given that only 30.2% of the participants obtained a CWL, the benefits of the comprehensive lifestyle intervention involved in the current study seem to be limited and thus they need to be reinforced.

Taken together, the findings of the present study highlight the importance of monitoring and dealing with exceedingly high levels of autonomy and imbalance among psychological well-being dimensions during a lifestyle intervention aimed at improving weight outcomes, especially among patients who showed poor response to treatment.

This study presents some limitations. First, given that some of the patients agreed to join the behavioral lifestyle intervention several months before the beginning of the program, they might have started dieting and doing exercise by themselves in the meantime. It is unknown whether this would have influenced the results of the present research. Second, the present study included a single cohort without a control group (such as a waiting list). Hence, it is difficult to discern to what extent the changes of the dependent variables were ascribable to lifestyle intervention only. Third, the present investigation measured the changes that occurred from pre- to post-treatment. Therefore, future studies assessing the longitudinal associations of psychological well-being dimensions with weight outcomes in the mid-and long-term, are needed. Fourth, diet and physical activity were evaluated using a self-rated questionnaire, due to its simple feasibility in a busy clinical setting (Prince et al., 2008). It is thus possible that the self-rated scores on diet and exercise were higher or lower than their actual levels. Finally, in the present study three quarters of the sample were women. This may limit the generalizability of our findings. Indeed, women with unfavorable psychological conditions are more likely to seek medical support for weight control (Kim et al., 2021), and females with obesity seem to be disappropriately affected by body image-related problems that may be linked to negative health outcomes (Dalle Grave et al., 2020; Weinberger, Kersting, Riedel-Heller, & Luck-Sikorski, 2016). Further studies are warranted to address these issues.

The results of this study have the potential to overcome some gaps in the literature and to provide suggestions for clinical practice. The present findings unraveled the role of psychological well-being in achieving clinically relevant weight loss. Unbalanced psychological well-being, in particular increasing autonomy, may contribute to a poor weight loss outcome. Future studies should consider psychological well-being as a mediator of the effect of behavioral lifestyle interventions on weight loss. When providing this kind of intervention, clinicians should be aware of the role of psychological well-being, which may hinder or strengthen weight loss. Psychotherapeutic approaches, such as Well-Being Therapy (Fava, 2016; Guidi et al., 2018), aimed at promoting balanced levels of psychological well-being and achieving a state of euthymia, may be promising new additions to lifestyle intervention for obesity.
